# ﻿First morphological description of the larval stages of three *Microtendipes* species (Diptera, Chironomidae) from South China with molecular confirmation

**DOI:** 10.3897/zookeys.1255.161652

**Published:** 2025-10-09

**Authors:** Haobo Jin, Yiyi Wang, Chao Song, Xin Qi

**Affiliations:** 1 College of Life Sciences, Taizhou University, Taizhou, Zhejiang 318000, China Taizhou University Taizhou China

**Keywords:** DNA barcodes, freshwater biomonitoring, identification key, larvae, *
Microtendipes
*, taxonomy

## Abstract

This study presents the first integrated morphological and molecular characterization of larvae from three *Microtendipes* species, *Microtendipes baishanzuensis* Song & Qi, 2023, *Microtendipes robustus* Song & Qi, 2023 and *Microtendipes tuberosus* Qi & Wang, 2006, collected from subtropical streams in China, providing important insights for advancing Chironomidae taxonomy. Using detailed morphometric analysis (head capsule ratios, mandibular pecten length, and striae counts) in conjunction with mitochondrial COI barcoding, we established larval–adult associations and differentiated these species from their congeners. *Microtendipes baishanzuensis* is characterized by a brownish head capsule with distinctly paler median teeth compared to the lateral teeth, the longest mandibular pecten within the genus, the highest number of body striae, and an exceptionally anteriorly positioned ring organ. *Microtendipes robustus* is distinguished by a uniformly dark brown mentum, remarkably wide ventromental plates, the most variable striae count, and a medium-sized mandibular pecten. *Microtendipes tuberosus* exhibits a uniformly dark brown mentum with median teeth conspicuously shorter than the second lateral teeth, the smallest body size in the genus, the shortest mandibular pecten, the fewest striae, and the most posteriorly located ring organ. A revised larval key for Chinese *Microtendipes* is presented, improving freshwater biomonitoring and addressing challenges associated with cryptic diversity.

## ﻿Introduction

The genus *Microtendipes* Kieffer was established by Kieffer in 1915 and currently comprises over 60 described species (Freeman and Cranston 1980; Cranston and Martin 1989; Ashe and Cranston 1990; Oliver et al. 1990; Zorina 2001; Qi and Wang 2006; Qi et al. 2014; Yamamoto and Yamamoto 2014; Hazra et al. 2016; Tang and Hiromi 2017; Song et al. 2023). In China, 18 recorded species have been described (Qi and Wang 2006; Qi et al. 2012, 2014; Song et al. 2023). Taxonomic research on *Microtendipes* has primarily focused on adult morphology, and larval descriptions are scarce. This gap is largely due to the high morphological homogeneity of larvae and the lack of reliable diagnostic traits, particularly distinct autapomorphies. These limitations collectively hinder species differentiation, precise taxonomic classification, and the establishment of a robust larval classification system. Furthermore, significant technical challenges persist in specimen collection and analysis, requiring specialized apparatus and microscopic techniques, and database limitations hinder reliable larva–adult matching. Despite these challenges, DNA barcoding has emerged as an effective identification tool (Carew et al. 2005, 2007; Gadawski et al. 2022).

DNA barcodes are included in most new species descriptions as a standard method for delimiting non-biting midges (Song et al. 2016, 2022; Yan et al. 2017; Lin et al. 2019). DNA barcoding has also proven effective for associating specimens across different life stages (Song et al. 2018). However, the larval stages of these species have remained undiscovered. To address this gap in the literature, we conducted extensive larval collections and performed DNA barcode analyses. Consequently, we successfully collected and identified the larvae of three *Microtendipes* species: *M. baishanzuensis* Song & Qi, 2023, *M. robustus* Song & Qi, 2023, and *M. tuberosus* Qi & Wang, 2006, providing the first descriptions of their larval stages.

## ﻿Material and methods

Larval specimens were collected from multiple rivers and streams using D-shaped nets. All specimens were preserved in 75% ethanol and stored at −20 °C in the laboratory until subsequent morphological and molecular analyses. For morphological examination, specimens were slide-mounted in Euparal and examined under a microscope. Measurements followed standardized protocols, and morphological terminology and abbreviations were adopted from Sæther (1980) and Maschwitz and Cook (2000). Values are reported as ranges accompanied by mean values, with the number of observed specimens (*N*) indicated in parentheses. All specimens are deposited in the College of Life Sciences, Taizhou University.

Genomic DNA was extracted using the protocol outlined by Song et al. (2018). The standard barcode region of the 5′ portion of the mitochondrial gene cytochrome *c* oxidase I (COI-5P) was amplified using the universal primers LCO1490 and HCO2198 (Folmer et al. 1994). PCR amplifications were performed as described by Song et al. (2018). PCR products were electrophoresed on a 1.0% agarose gel, purified, and sequenced using an ABI 3730XL capillary sequencer (Beijing Genomics Institute Co., Ltd., Hangzhou, China). Raw sequences were edited using BioEdit v. 7.2.5 (Hall 1999).

Publicly available *Microtendipes* sequences (≥500 bp in length) were retrieved from the Barcode of Life Data System (BOLD; http://www.boldsystems.org/) on June 20, 2024 (see Suppl. material 1 for details). Sequence alignment was conducted in MEGA v. 12 (Kumar et al. 2024) using the ClustalW algorithm. Pairwise genetic distances were calculated using the K2P model in MEGA v. 12. A neighbor-joining (NJ) tree was constructed based on the Kimura 2-Parameter (K2P) substitution model with 1000 bootstrap replicates, using the “pairwise deletion” option to handle missing data. A maximum-likelihood (ML) tree was generated using IQ-TREE v. 2.1.3 (Nguyen et al. 2015), with node support assessed through ultrafast bootstrapping with 1000 replicates. Bayesian inference (BI) analysis was conducted using MrBayes v. 3.2.7 (Ronquist et al. 2012), employing Markov chain Monte Carlo (MCMC) randomization for 10 million generations, with the first 25% of trees discarded as burn-in. Trace files from the BI analysis were examined using Tracer v. 1.7 (Rambaut et al. 2018), and the final phylogenetic tree was visualized in FigTree v. 1.4.2.

### ﻿Abbreviations

Morphological abbreviations of larval characters are used as follow: Ant 1–6, length of antennal segments 1–6 in µm; A1R, first antennal segment ratio, length of segment 1: width of segment 1 through the ring organ; AR, antennal ratio, length of basal segment: combined lengths of segments 2 to apex; B. l., total body length in mm; Bl, antennal blade length; BlR, blade ratio, length of blade: total length of Ant 2–6; Pmd, premandible length; H. l., head capsule length, length from anterior labrum to posterior margin of capsule; L, larva; Md, mandible length; M. w, mentum width; Mmw, median mental tooth width; PM, postmentum length; ROR, ring organ ratio, distance from basal to location of ring organ: length of basal antennal segment; SAS, length of supraanal seta; SSm–SSm: distance between setae submenti; V. w, ventromentum width; V. l, ventromentum length; IPD, inter-ventromentum plates distance; Str, striae (Sæther 1980; Maschwitz and Cook 2000).

## ﻿Results and discussion

### ﻿DNA barcodes analysis

**Figure 1.**
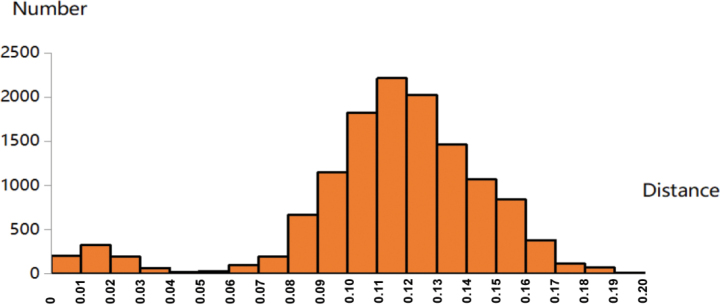
Histogram of pairwise K2P distances between morphological species of *Microtendipes*. The horizontal axis represents the pairwise K2P distance, the vertical axis represents the number of pairwise sequence comparisons.

This study analyzed a total of 161 sequences, including eight newly obtained larval sequences. A clear barcode gap of 4–6% was observed (Fig. 1), confirming the effectiveness of DNA barcoding for species delimitation within this genus. Based on three known male specimens, the maximum intraspecific genetic distance was 0.7% (mean: 0.5%) for *M. baishanzuensis*, 5.8% (mean: 2.9%) for *M. robustus*, and 3.2% (mean: 1.4%) for *M. tuberosus*. After including larval DNA sequences, these values changed slightly: *M. baishanzuensis* exhibited a maximum of 0.9% (mean: 0.5%), *M. robustus* a maximum of 5.8% (mean: 2.5%), and *M. tuberosus* a maximum of 6.1% (mean: 2.6%) (Suppl. material 2). Notably, the maximum intraspecific distance observed in *M. tuberosus* (6.1%) remained below the minimum interspecific distance (8.0%) between *M. baishanzuensis* and *M. robustus*.

**Figure 2.**
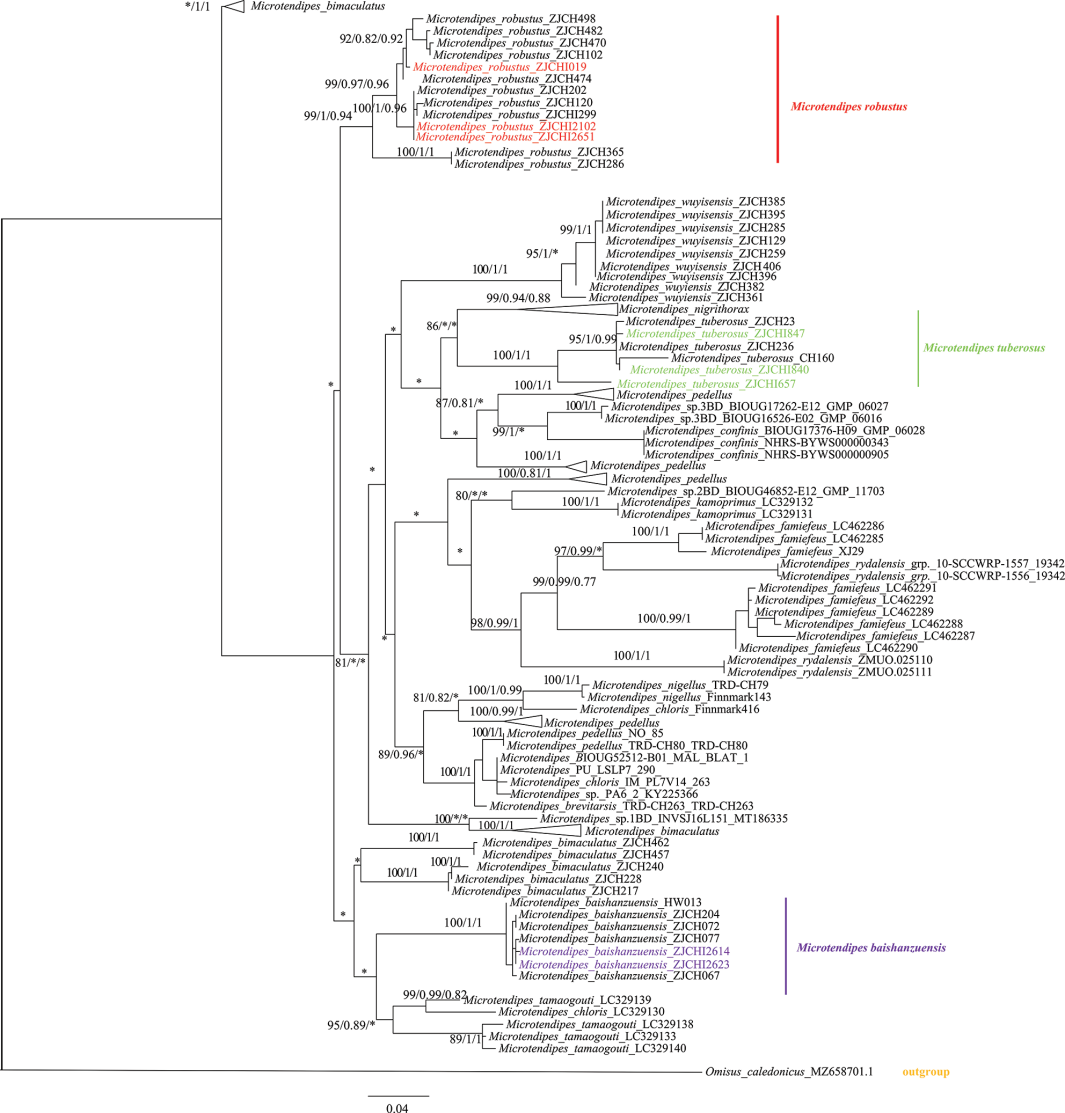
Phylogeny tree for *Microtendipes* based on DNA barcode sequences. The tree was based on partial COI sequences and the generalized time-reversible substitution model. *Omisus caledonicus* (Edwards) was used as an outgroup. The phylogenetic analysis incorporated data from maximum-likelihood (ML), Bayesian inference (BI), and neighbor-joining (NJ) methods for all three identified species; the data are presented as ML/BI/NJ analyses. Only nodes with Ultra-BS (ML) > 80%, PP > 0.80, and BS(NJ) > 0.75 are shown; different colors within each area represent larvae.

Phylogenetic analyses (Neighbor-joining, maximum likelihood, and Bayesian inference) revealed strong concordance between larval and adult sequences (Fig. 2). For *M. tuberosus*, although minor topological variations occurred across trees, all three methods provided robust support for the same clade structure. In *M. robustus*, consistent results across all three methods (NJ/ML/BI) confirmed larval–adult conspecificity via DNA barcoding. For *M. baishanzuensis*, all three phylogenetic reconstructions yielded well-resolved, congruent clades. This high consistency in branching patterns, combined with the low maximum pairwise genetic distance (0.9%), conclusively identifies the larvae as *M. baishanzuensis* using DNA barcoding.

### ﻿Taxonomy

#### ﻿ Microtendipes baishanzuensis

Taxon classificationAnimaliaDipteraChironomidae

Song & Qi, 2023

CA90D76E-C0CE-514E-AF3A-E16EF1C09F9C

Figs 3B–E, 4

Microtendipes baishanzuensis Song & Qi, 2023: 8.

##### Material examined

**(*N* = 2)**: 2 larvae, China, Zhejiang Province, Lishui City, Qingyuan County, Baishanzu Town, 1600 m a.s.l., 27.750°N, 119.198°E, 14.VIII.2020, C. Song.

##### Diagnosis

**Figure 3.**
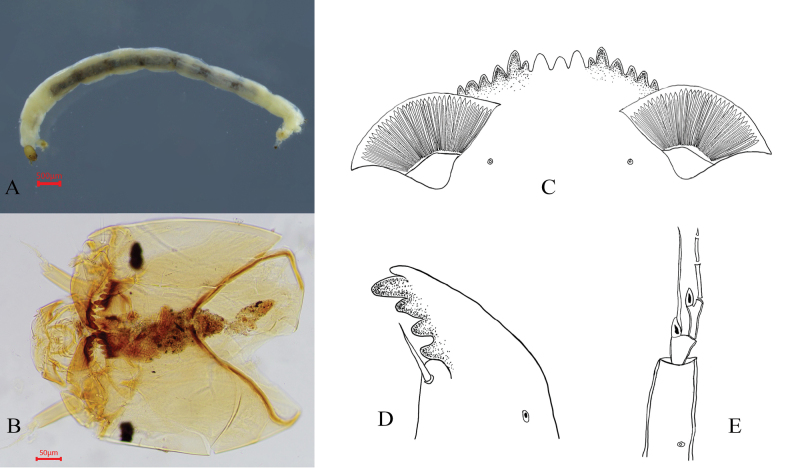
Larva of *Microtendipes baishanzuensis* Song & Qi, 2023. A. Habitus of larva; B. Head capsule; C. Mentum; D. Mandible; E. Antenna.

(Fig. 3B, C). Mentum dark brown, with median teeth distinctly paler than lateral teeth; cephalic capsule brown. Total length 7.1–7.8, 7.5 mm; head capsule length 490.0–520.0, 505.0 μm; head capsule width 450.0–460.0, 455.0 μm.

Mentum (Fig. 3C). Width 152.5 μm; median tooth width 37.5 μm; ventromental plate width 125.0–132.5, 128.8 μm; distance between ventromental plates 83.0–90.0, 86.5 μm; striae count 35.0–40.0, 37.5.

Mandible (Fig. 3D). Length 168.0–195.0, 181.5 μm.

Antenna (Fig. 3E). Segment lengths, 102.5–112.5, 107.5 μm; 25.0–27.5, 26.3 μm; 27.0 μm; 25.0 μm; 17.0–17.5,17.3 μm; 10.0 μm. Antennal ratio (AR) 1.0–1.1, 1.0. Ring organ width 34.0–35.0, 34.5 μm; ring organ positioned at 0.2–0.3, 0.3 of basal segment length, 22.5–35.0, 28.8 μm from segment base; antennal blade 106.0–125.0, 115.5 μm in length, width 2.0–18.0 μm.

Labrum. Premandible with two apical teeth; length 97.5–120.0, 108.8 μm. Pecten epipharyngis with three broad marginal teeth apically; labral lamella composed of 15 small teeth.

Thorax. Anterior claws pale with a little golden posterior claws pale golden, both simple and dense.

Abdomen. 8 tail hairs of anal seta, 520.0–611.0, 565.5 μm (Table 1).

**Table 1.** Mensural features of the larvae of *Microtendipes baishanzuensis*, *Microtendipes tuberosus* and *Microtendipes robustus*. Abbreviations follow as in the text.

	M. baishanzuensis	M. tuberosus	M. robustus
*N*	2	3	3
B.l. /mm	7.1–7.8, 7.5	2.1–3.7, 2.8	2.9–9.6, 5.0
H. w.	450–460, 455	287.5–300, 292.5	287.5–620.0, 480.6
PM	200–202.5, 201.25	150–157.5, 153.3	125–228, 190.8
SSm–SSm	105–100, 102.5	67.5–77.5, 72.1	71–128, 109.8
Ant1	102.5–112.5, 107.5	100–102.5, 101.2	42.5–117.5, 75.8
Ant2	25–27.5, 26.3	27–28, 27.5	15.5–25, 22.6
Ant3	27.0	23–26.3, 24.8	17.5–27.5, 21.9
Ant4	25.0	16–21.3, 19.1	15–18, 17.1
Ant5	17–17.5, 17.3	10–11.3, 10.4	11.0–15.0, 12.9
Ant6	10.0	7.5–8.8, 7.9	5.0–8.0, 6.1
AR	1.0–1.1, 1.0	1.1–1.2, 1.1	0.8–1.3, 0.9
AIR	3.0–3.2, 3.1	3.7–4.4,4.0	1.4–3.2, 2.5
ROR	0.2–0.3, 0.27	0.2–0.2, 0.2	0.3–0.7, 0.4
Bl	106–125, 115.5	53–72, 59.67	87.5–137.5, 114.4
BlR	1.0–1.2, 1.1	0.6–0.8, 0.7	1.3–1.6, 1.4
Pmd	97.5–120, 108.8	62.5–65, 63.8	60–110, 90
Md	168–195, 181.5	125–132.5, 129.8	115–200, 174.4
M. w	152.5–152.5, 152.5	105–110, 108	92.5–185, 151.9
Mmw	37.5	23–25, 24.3	20.5–42.5, 33.3
AS	520–611, 565.5	197.4–445, 327.5	347–602.3, 492.5
V. w	125–132.5, 128.8	83–122.5, 96.8	75–150, 120
V. l	75–80, 77.5	37.5–57.5, 49.3	48–87.5, 72.3
IPD	83–90, 86.5	59–85, 68.8	51–125, 105.9
Str	35–40, 37.5	22–29, 26	28–43, 38.3
SAS	8.0–10.0, 9.0	8.5–9.5, 9.0	8.5–12.5, 10.3

##### Ecological environment

**Figure 4.**
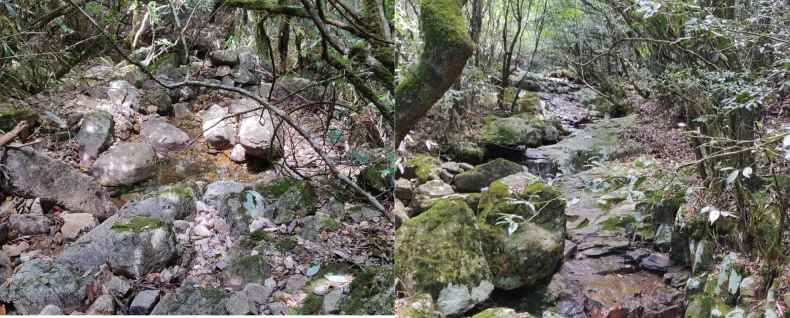
Habitat of *Microtendipes baishanzuensis* larvae.

(Fig. 4). A forest stream, winding through wooded areas, exhibiting high-clarity water flowing over a predominantly rocky substrate.

##### Remarks.

A comparative analysis was conducted between our data and the larval morphological measurements reported by Tang (2006). The larvae of *M. baishanzuensis* resemble *Microtendipes pedellus* (De Geer, 1929) and *Microtendipes chloris* (Meigen, 1933) in the shape of the mentum. However, *M. baishanzuensis* has more striae than *M. pedellus* (mean 37.5 vs. 30.0). The total length of the postmentum in *M. baishanzuensis* is shorter than in *M. pedellus* (mean 201.3 μm vs. 223.0 μm). The ring organ in *M. baishanzuensis* is located at 0.2–0.3 (mean 0.267) of the basal segment, compared to approximately 0.33. Compared to *M. chloris*, *M. baishanzuensis* has a distinctly shorter blade (mean 115.5 μm vs. 145.0 μm), a longer postmentum (mean 201.3 μm vs. 215.0 μm), and more striae (mean 37.5 vs. 28.0).

#### ﻿ Microtendipes tuberosus

Taxon classificationAnimaliaDipteraChironomidae

Qi & Wang, 2006

80E993F8-5CDF-5499-A18D-925216EB36C6

Figs 5B–E, 6

Microtendipes tuberosus Qi & Wang, 2006: 43.

##### Material examined

**(*N* = 3)**: 1 larva, China, Zhejiang Province, Lishui City, Suichang County, Jiulong Mountain National Nature Reserve, 370 m a.s.l., 28.408°N, 118.811°E, 29.VIII. 2020, C. Song; 2 larvae, China, Zhejiang Province, Lishui City, Suichang County, Jiulong Mountain National Nature Reserve, 400 m a.s.l., 28.380°N, 118.799°E, 29.VIII. 2020, C. Song.

##### Diagnosis

**Figure 5.**
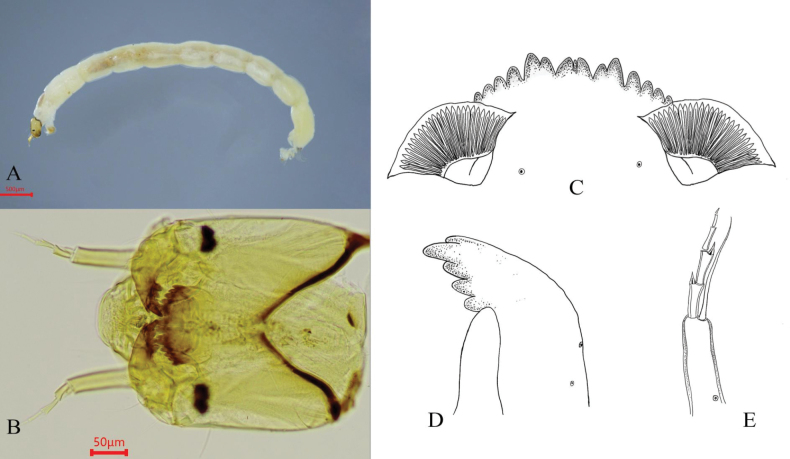
Larva of *Microtendipes tuberosus* Qi & Wang, 2006. A. Habitus of larva; B. Head capsule; C. Mentum; D. Mandible; E. Antenna.

(Fig. 5B, C). Mentum dark brown, with median teeth concolorous with lateral teeth; median tooth slightly shorter than the second lateral tooth. The cephalic capsule is light brown. Total length 2.1–3.7, 2.8 mm; head capsule length 375.0–400.0, 387.5 μm; head capsule width 287.5–300.0, 292.5 μm.

Mentum (Fig. 5C). Width 105.0–110.0, 108.0 μm; median tooth width 23.0–25.0, 24.3 μm; ventromental plate width 83.0–122.5, 96.8 μm; distance between ventromental plates 59.0–85.0, 68.8 μm; striae count 22–29, 26.

Mandible (Fig. 5D). Length 125.0–132.5, 129.8 μm.

Antenna (Fig. 5E). Segment lengths: 100.0–102.5, 101.2 μm; 27.0–28.0, 27.5 μm; 23.0–26.3, 24.8 μm; 16.0–21.3, 19.1 μm; 10.0–11.3, 10.4 μm; 7.5–8.8, 7.9 μm).

Antennal ratio (AR) 1.1–1.2, 1.1. Ring organ width 25.5 μm; ring organ positioned at 0.15–0.22, 0.20 of basal segment length, 15.0–22.5, 20.0 μm from segment base; antennal blade 53.0–72.0, 59.7 μm in length.

Labrum. Premandible bifid; length 62.5–65.0, 63.8 μm. Pecten epipharyngis is composed of 8 teeth.

Thorax. Anterior claws pale with a faint golden tint; posterior claws pale golden. Procercus and apical setae are yellowish brown.

Abdomen. Anal setae comprising 8 hairs, length 197.4–445.0, 327.5 μm (Table 1).

##### Ecological environment

**Figure 6.**
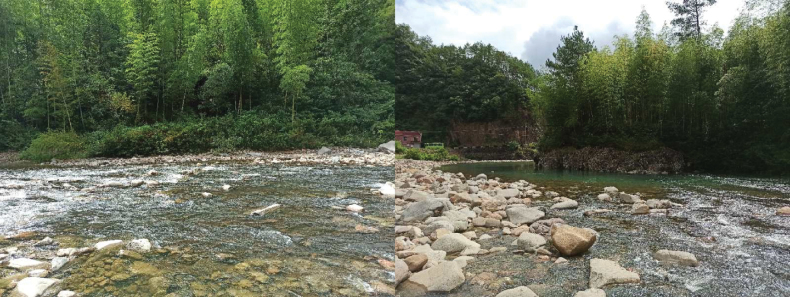
Habitat of *Microtendipes tuberosus* larvae.

(Fig. 6). The stream flows through forests and villages, featuring a streambed composed of bedrock and sediment, with excellent water clarity.

##### Remarks.

A comparative analysis was conducted between our data and the larval morphological measurements reported by Tang (2006). The larvae of *M. tuberosus* resemble those of *Microtendipes britteni* (Edwards, 1983) in mentum morphology. However, *M. tuberosus* is noticeably smaller in body size (mean 2.8 mm vs. 6.9 mm in *M. britteni*) and has a shorter mandibular pecten (mean 153.3 μm vs. 195.0 μm in *M. britteni*). Among congeners, *M. tuberosus* is notably smaller overall. Both its median and lateral teeth are uniformly dark brown.

#### ﻿ Microtendipes robustus

Taxon classificationAnimaliaDipteraChironomidae

Song & Qi, 2023

A6D2358A-FD95-56BB-BD27-18BC432B3B56

Figs 7B–E, 8

Microtendipes robustus Song & Qi, 2023:16.

##### Material examined

**(*N* = 3)**: 1 larva, China, Zhejiang Province, Lishui City, Qingyuan County, Baishanzu Town, 1370 m, 27.754°N, 119.186°E, 14.VIII.2020, C. Song; 1 larva, China, Zhejiang Province, Lishui City, Qingyuan County, Baishanzu Town, 1650 m a.s.l., 27.751°N, 119.199°E, 14.VIII.2020, C. Song; 1 larva, China, Zhejiang Province, Wenzhou City, Taishun County, Wuyanling National Nature Reserve, 1060 m a.s.l., 27.424°N, 119.404°E, 25.IX.2020, C. Song; 1 larva, China, Zhejiang Province, Wenzhou City, Taishun County, Wuyanling National Nature Reserve, 660 m a.s.l., 27.423°N, 119.404°E, 25.IX.2020, C. Song.

##### Diagnosis

**Figure 7.**
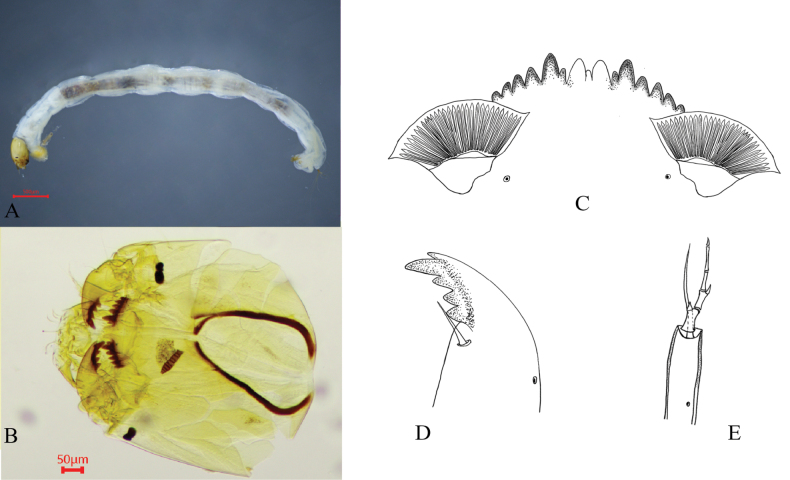
Larva of *Microtendipes robustus* Song & Qi, 2023. A. Habitus of larva; B. Head capsule; C. Mentum; D. Mandible; E. Antenna.

(Fig. 7B, C). The larvae possess a light brown cephalic capsule and dark brown mentum, with the pale median teeth forming a distinct contrast against the darker lateral teeth.

Total length. 2.9–9.6 mm, 5.0 mm; head capsule length 325.0–650.0, 521.3 μm; head capsule width 287.5–620.0, 480.6 μm.

Mentum (Fig. 7C). Width 92.5–185.0, 151.9 μm; median tooth width 20.5–42.5, 33.3 μm; ventromental plate width 75.0–150.0, 120.0 μm; distance between ventromental plates 51.0–125.0, 105.9 μm; number of striae 28–43, 38.3.

Mandible (Fig. 7D). Length 115.0–200.0, 174.4 μm.

Antenna (Fig. 7E). Segment lengths, 42.5–117.5, 75.8 μm; 15.5–25.0, 22.6 μm; 17.5–27.5, 21.9 μm; 15.0–18.0, 17.1 μm; 11.0–15.0, 12.9 μm; 5.0–8.0, 6.1 μm. Antennal ratio (AR) 0.8–1.3, 0.9. Ring organ width 20.0–37.0, 30.5 μm; ring organ positioned at 0.3–0.6, 0.4 of basal segment, 22.5–32.5, 26.3 μm from segment base.

Labrum. Premandible with two apical teeth; length 60.0–110.0, 90.0 μm. Pecten epipharyngis with three broad marginal teeth apically; labral lamella composed of 16–17 small teeth.

Thorax. Anterior claws pale with golden hints; posterior claws darker, pale brown. Procercus and apical setae are yellowish brown.

Abdomen. 8 tail hairs of anal seta, 347.0–602.3, 492.5 μm (Table 1).

##### Ecological environment

**Figure 8.**
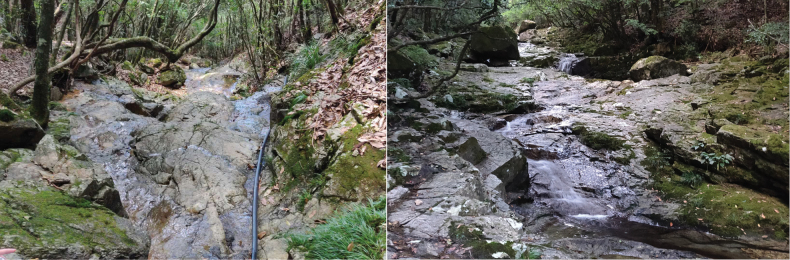
Habitat of *Microtendipes robustus* larvae.

(Fig. 8). A mountain stream, fed by natural springs, meandering through forested terrain with a bedrock-dominated streambed and pristine water.

##### Remarks.

A comparative analysis was conducted between our data and the larval morphological measurements reported in Tang (2006). The larvae of *M. robustus* are similar to those of *M. pedellus* in mentum shape. However, *M. robustus* has a shorter overall body length (mean 5.0 mm vs. 8.8 mm in *M. pedellus*), shorter postmentum (mean 190.8 μm vs. 223.0 μm in *M. pedellus*), as well as a shorter mentum (mean 174.4 μm vs. 210.0 μm in *M. pedellus*) compared to *M. pedellus*.

## ﻿Conclusions

*Microtendipes baishanzuensis* resembles *M. pedellus* and *M. chloris* in mentum shape but differs by having more striae and a longer postmentum than *M. pedellus*, as well as a larger body size and more striae than *M. chloris*. *Microtendipes tuberosus* is generally similar to congeners but is notably smaller in overall size and has a shorter postmentum than *M. britteni*. *Microtendipes robustus* is challenging to identify because of its morphological overlap with *M. pedellus*, but key distinguishing characteristics include a shorter ventromental plate length and a greater plate distance. These specific morphometric differences, combined with molecular evidence, provide reliable criteria for species identification within this taxonomically complex genus.

### ﻿Key to known larvae of *Microtendipes* Kieffer, modified from Tang (2006)

**Table:** 

1	Mental plate lacking distinct median teeth; anterior margin straight. Median teeth subequal in size; all teeth on the mental plate dark brown. Distributed in Afrotropical and Oriental regions	***M. numerosus* Lehmann**
–	Mental plate with three characteristic median teeth, equal to or slightly lower than second lateral teeth; bases of 1^st^ and 2^nd^ lateral teeth fused	**2**
2	Median teeth distinct, always paler than lateral teeth and equal/subequal in size (if equal at base, median teeth slightly smaller); pecten epipharyngis single, with 3–4 large central teeth and 2–3 smaller lateral teeth; premandible with 5 teeth	**3**
–	Median teeth trifid, central median tooth often reduced/indistinct; all three median teeth paler or concolorous with lateral teeth; premandible with 3 teeth	**5**
3	Central median tooth slightly wider than or equal to outer median teeth; blade reaching apex of fourth antennal segment; labral lamella with 12–14 small teeth; pecten epipharyngis comprising 3–4 large and 3–5 small teeth	***M. rydalensis* (Edwards)**
–	Central median tooth smaller than outer lateral teeth	**4**
4	Pecten epipharyngis bearing three broad apical marginal teeth; labral lamella with 20–22 small teeth; blade significantly longer than flagellum	***M. tarsalis* (Walker)**
–	Pecten epipharyngis with 9–11 teeth (mixed large and small); labral lamella with 22–24 small teeth; blade slightly shorter than or equal to flagellum	***M. truncatus* Kawai & Sasa**
5	Median teeth dark brown, concolorous with lateral teeth	**6**
–	Median teeth distinctly paler than other teeth; pecten epipharyngis often trilobed	**7**
6	Labral lamella with 14–20 fine teeth	**8**
–	Labral lamella with 12–16 teeth	**9**
7	Pecten epipharyngis with three broad marginal teeth apically; premandible with two apical teeth (length 60.0–110.0 μm, mean 90.0); labral lamella with 16–17 teeth; cephalic capsule light brown, mentum dark brown with pale median teeth	***M. robustus* Song & Qi**
–	Pecten epipharyngis not with three broad marginal teeth apically; premandible with three teeth	**10**
8	Pecten epipharyngis with three broad marginal teeth apically; premandible with two apical teeth (length 97.5–120.0 μm, mean 108.8); labral lamella with 15 teeth	***M. baishanzuensis* Song & Qi**
–	Pecten epipharyngis not with three broad marginal teeth; premandible not bifid; striae: 28–32 (mean 30); head width: 360–410 μm (mean 380)	***M. pedellus* (De Geer)**
9	Premandible bifid (length 62.5–65.0 μm, mean 63.8); pecten epipharyngis with 8 teeth; total length: 2.1–3.7 mm (mean 2.8)	***M. tuberosus* Qi & Wang**
–	Premandible not bifid; pecten epipharyngis not with 8 teeth; total length: 5.5–9.4 mm (mean 6.9)	***M. britteni* (Edwards)**
10	Labral lamella with 14–18 teeth (mode 16)	***M. diffinis* Edwards**
–	Labral lamella with 19–22 teeth (mode 20)	***M. chloris* Meigen**

## Supplementary Material

XML Treatment for Microtendipes baishanzuensis

XML Treatment for Microtendipes tuberosus

XML Treatment for Microtendipes robustus

## ﻿Additional information

### Use of AI

No use of AI was reported.

### Data availability

All of the data that support the findings of this study are available in the main text or Supplementary Information.

## Supplementary materials

Supplementary material 1 Publicly available *Microtendipes* sequences Data type xls https://binary.pensoft.net/file/1433995This dataset is made available under the Open Database License (http://opendatacommons.org/licenses/odbl/1.0/). The Open Database License (ODbL) is a license agreement intended to allow users to freely share, modify, and use this Dataset while maintaining this same freedom for others, provided that the original source and author(s) are credited.Haobo Jin, Yiyi Wang, Chao Song, Xin Qi

Supplementary material 2 DNA barcodes analysis Data type xls https://binary.pensoft.net/file/1433996This dataset is made available under the Open Database License (http://opendatacommons.org/licenses/odbl/1.0/). The Open Database License (ODbL) is a license agreement intended to allow users to freely share, modify, and use this Dataset while maintaining this same freedom for others, provided that the original source and author(s) are credited.Haobo Jin, Yiyi Wang, Chao Song, Xin Qi

## References

[B1] Ashe P Cranston PS (1990) Family Chironomidae. In: Soós Á Papp L (Eds) Catalogue of Palaearctic Diptera, Vol.2. Akadémiai Kiadó, Budapest, 113–499.

[B2] Carew ME Pettigrove V Hoffmann AA (2005) The utility of DNA markers in classical taxonomy: Using Cytochrome Oxidase I markers to differentiate Australian *Cladopelma* (Diptera: Chironomidae) midges. Annals of the Entomological Society of America 98(4): 587–594. 10.1603/0013-8746(2005)098[0587:TUODMI]2.0.CO;2

[B3] Carew ME Pettigrove V Cox RL Hoffmann AA (2007) DNA identification of urban Tanytarsini chironomids (Diptera: Chironomidae). Journal of the North American Benthological Society 26(2): 587–600. 10.1899/06-120.1

[B4] Cranston PS Martin J (1989) Family Chironomidae. In: Evenhuis NL (Ed.) Catalog of the Diptera of the Australasian and Oceanian Regions.Bishop Museum Press, Honolulu and Brill, E.J., Leiden, 252–274.

[B5] Folmer O Black M Hoeh W Lutz R Vrijenhoek R (1994) DNA primers for amplification of mitochondrial cytochrome c oxidase subunit I from diverse metazoan invertebrates. Molecular Marine Biology and Biotechnology 3: 294–299. 7881515

[B6] Freeman P Cranston PS (1980) Family Chironomidae. In: Crosskey RW (Ed.) Catalogue of the Diptera of the Afrotropical region.British Museum (Natural History), London, 175–202.

[B7] Gadawski P Montagna M Rossaro B Gilka W Pesic V Grabowski M Magoga G (2022) DNA barcoding of Chironomidae from the Lake Skadar region: Reference library and a comparative analysis of the European fauna. Diversity & Distributions 28(12): 2838–2857. 10.1111/ddi.13504

[B8] Hall TA (1999) BioEdit: A user-friendly biological sequence alignment editor and analysis program for Windows 95/98/NT. Nucleic Acids Symposium Series 41: 95–98. 10.1021/bk-1999-0734.ch008

[B9] Hazra N Niitsuma H Chaudhuri PK (2016) Checklist of chironomid midges (Diptera: Chironomidae) of the Oriental Region (Occasional Paper No. 376). Zoological Survey of India, Kolkata, 138 pp.

[B10] Kieffer JJ (1915) Neue Chironomiden aus Mitteleuropa. Brotéria Série Zoológica 13: 65–87.

[B11] Kumar S Stecher G Suleski M Sanderford M Sharma S Tamura K (2024) MEGA12: Molecular Evolutionary Genetic Analysis Version 12 for Adaptive and Green Computing. Molecular Biology and Evolution 41(12): 263. 10.1093/molbev/msae263 PMC11683415 39708372

[B12] Lin XL Yu HJ Zhang RL Wang XH (2019) Polypedilum (Cerobregma) heberti sp. n. (Diptera: Chironomidae) from Gaoligong Mountains, Yunnan, China. Zootaxa 4571(2): 255–262. 10.11646/zootaxa.4571.2.5 31715818

[B13] Maschwitz DE Cook EF (2000) Revision of the Nearctic species of the genus *Polypedilum* Kieffer (Diptera: Chironomidae) in the subgenera P. (Polypedilum) Kieffer and P. (Uresipedilum) Oyewo and Sæther. Ohio Biological Survey, Columbus, 135 pp.

[B14] Nguyen LT Schmidt HA von Haeseler A Minh BQ (2015) IQ-TREE: A fast and effective stochastic algorithm for estimating maximum-likelihood phylogenies. Molecular Biology and Evolution 32(1): 268–274. 10.1093/molbev/msu300 25371430 PMC4271533

[B15] Oliver DR Dillon ME Cranston PS (1990) A Catalog of Nearctic Chironomidae. Research Branch, Agriculture Canada, Ottawa, 89 pp.

[B16] Qi X Wang XH (2006) A review of *Microtendipes* Kieffer from China (Diptera: Chironomidae). Zootaxa 1108(1): 37–51. 10.11646/zootaxa.1108.1.3

[B17] Qi X Lin XL Wang XH (2012) A new species of the genus *Microtendipes* Kieffer, 1915 (Diptera, Chironomidae) from Oriental China. ZooKeys 212: 81–89. 10.3897/zookeys.212.3329 PMC3428705 22933851

[B18] Qi X Li YF Wang XH Shao QJ (2014) A new species of *Microtendipes* (Diptera: Chironomidae) with a median volsella from Xishan Island, China. The Florida Entomologist 97(3): 871–876. 10.1653/024.097.0344

[B19] Rambaut A Drummond AJ Xie D Baele G Suchard MA (2018) Posterior summarization in Bayesian phylogenetics using Tracer 1.7. Systematic Biology 67(5): 901–904. 10.1093/sysbio/syy032 29718447 PMC6101584

[B20] Ronquist F Teslenko M Van Der Mark P Ayres DL Darling A Höhna S Larget B Liu L Suchard MA Huelsenbeck JP (2012) MrBayes 3.2: Efficient *Bayesian* phylogenetic inference and model choice across a large model space. Systematic Biology 61(3): 539–542. 10.1093/sysbio/sys029 22357727 PMC3329765

[B21] Sæther OA (1980) Glossary of chironomid morphology terminology (Diptera: Chironomidae). Entomologica Scandinavica (Supplement 14): 1–51.

[B22] Song C Wang Q Zhang RL Sun BJ Wang XH (2016) Exploring the utility of DNA barcoding in species delimitation of Polypedilum (Tripodura) non-biting midges (Diptera: Chironomidae). Zootaxa 4079(5): 534–550. 10.11646/zootaxa.4079.5.2 27394207

[B23] Song C Lin XL Wang Q Wang XH (2018) DNA barcodes successfully delimit morphospecies in a superdiverse insect genus. Zoologica Scripta 47(3): 311–324. 10.1111/zsc.12284

[B24] Song C Zhu BQ Moubayed-Breil J Lei T Qi X (2022) Taxonomic study on the genus *Stenochironomus* Kieffer from the Baishanzu Nature Reserve, China (Diptera, Chironomidae). ZooKeys 1104: 93–113. 10.3897/zookeys.1104.81403 36761924 PMC9848771

[B25] Song C Wang L Lei T Qi X (2023) New color-patterned species of *Microtendipes* Kieffer, 1913 (Diptera: Chironomidae) and a Deep Intraspecific Divergence of Species by DNA Barcodes. Insects 14(3): 227. 10.3390/insects14030227 36975912 PMC10054112

[B26] Tang HQ (2006) Biosystematic study on the chironomid larvae in China (Diptera: Chironomidae). PhD Thesis, Nankai University, Tianjin, China.

[B27] Tang HQ Hiromi N (2017) Review of the Japanese *Microtendipes* (Diptera: Chironomidae: Chironominae), with description of a new species. Zootaxa 4320(3): 535–553. 10.11646/zootaxa.4320.3.8

[B28] Yamamoto M Yamamoto N (2014) Family Chironomidae. In: Editorial Committee of Catalogue of the Insects of Japan (Ed.) Catalogue of the Insects of Japan Vol.8, Diptera. Part 1: Nematocera-BrachyceraAschiza. Touka Shobo, Fukuoka, Japan, 237–362.

[B29] Yan CC Song C Liu T Zhao GJ Hou ZY Cao W Wang XH (2017) Two new and one newly recorded species of *Polypedilum* Kieffer 1912 with DNA barcodes from Oriental China (Chironomidae: Diptera). Zootaxa 4238(1): 109–118. 10.11646/zootaxa.4238.1.8 28264271

[B30] Zorina OV (2001) New species of the genera *Cryptotendipes*, *Dicrotendipes*, *Microtendipes* and *Stenochironomus* (Diptera, Chironomidae, Chironominae) from the Russian Far East. Vestnik Zoologii 35: 31–38.

